# Straining induced spontaneous bowel transection in a patient with incarcerated inguinal hernia with cryptorchidism: A case report

**DOI:** 10.1016/j.ijscr.2025.111093

**Published:** 2025-02-25

**Authors:** Rahul Jha, Samrat Shrestha, Bijay Raj Bhatta, Ramesh Prasad Upadhayay, Rishika Prasad

**Affiliations:** a Department of General Surgery, National Academy of Medical Sciences, Kathmandu, Nepal; b Nepal Medical College Teaching Hospital, Kathmandu, Nepal

**Keywords:** Case report, Coughing, Straining induced, Bowel transection, Incarcerated hernia, Darning repair

## Abstract

**Introduction:**

The pooled prevalence of inguinal hernia worldwide is reported as 7.7 %. Out of all inguinal hernia cases, 10 % of cases get incarcerated whereas strangulation occurs in 0.29 % to 2.9 % of cases. The mortality rate of strangulated inguinal hernia is 2.6 % to 9 %. Although cough-induced spontaneous bowel transection in incarcerated inguinal hernia has been reported, straining induced spontaneous bowel transection has never been reported in the literature.

**Case report:**

40 year male presented with irreducible swelling in right inguinoscrotal region, vomiting and unable to pass stool/flatus for 4 days. On examination, there was irreducible swelling at right inguinoscrotal region, separately palpable left testis and non-palpable right testis with signs of peritonitis. On inguinoscrotal exploration, the hernial sac contained 10 ml of toxic fluid with a viable but spontaneously transected ileal loop. So, hand-sewn ileoileal anastomosis with darning repair with right orchidectomy was done.

**Discussion:**

An intraluminal pressure of 150–260 mm of Hg is required for bowel transection. In incarcerated hernia, bowel loops are edematous, and repeated episodes of straining during defecation can lead to an elevation of intraluminal pressure up to 230 mm of Hg, which is enough to cause transection of the edematous bowel.

**Conclusion:**

In incarcerated hernia, since the bowels are edematous, even repeated episodes of coughing or straining can cause bowel transection. Untreated undescended testis is at higher risk of developing malignancy after 10 years of age. Similarly, restoration of fertility is not seen even on orchidopexy. So, orchidectomy is recommended in adult.

## Highlights

• In incarcerated hernia, bower transection after trauma is common.
• But in incarcerated hernia, bowel transection following non traumatic event like coughing or straining is rare.
• Intraluminal pressure of 150-260 mm of Hg is required for transection of bowel.
• In incarcerated hernia, since the bowels are edematous, even non traumatic events may cause transection of bowel.

## 1 Introduction

Inguinal hernia means protrusion of abdominal viscera through a defect in the inguinal region of anterior abdominal wall [1]. Patient developing inguinal hernia are found to have a higher proportion of collagen III (having less tensile strength) as compared to collagen I [2]. The pooled prevalence of inguinal hernia worldwide is reported as 7.7 % whereas the lifetime risk of inguinal hernia in male and female is 27 % and 3 % respectively [3]. Incarcerated hernia is irreducible with intact vascularity of hernia sac content whereas, in strangulated hernia, the vascularity is compromised [1]. Out of all inguinal hernia cases, 10 % cases get incarcerated [4] whereas strangulation occurs in 0.29 % to 2.9 % cases. The mortality rate of strangulated inguinal hernia is said to be 2.6 % to 9 % [5]. Although cough induced spontaneous bowel transection in incarcerated inguinal hernia has been reported, straining induced spontaneous bowel transection in incarcerated inguinal hernia has never been reported in the literature.

The work has been done in line with SCARE guidelines [6].

## 2 Case report

**Fig. 1:**
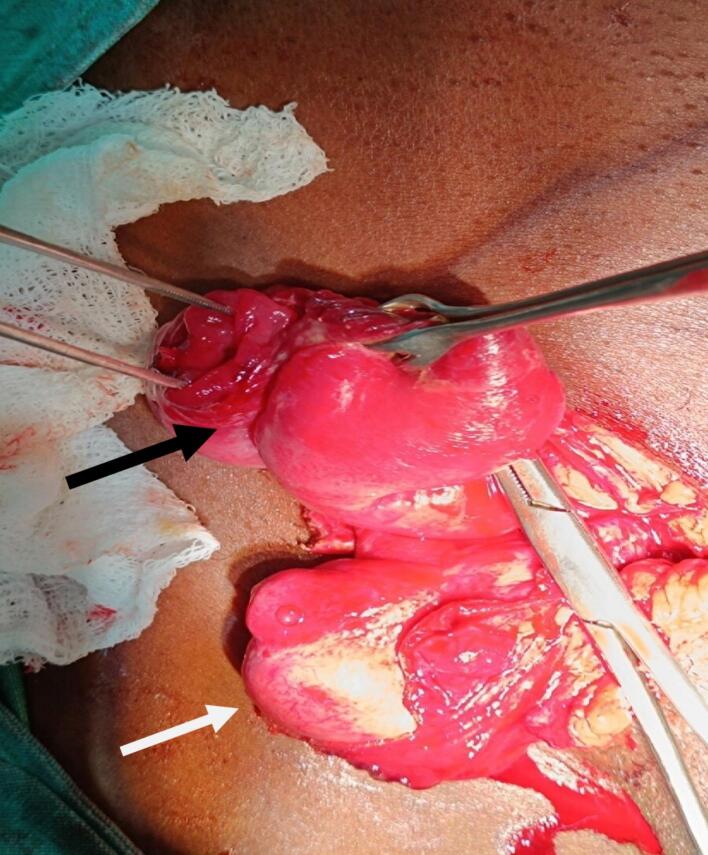
Black arrow showing straining induced spontaneously transected ileal loops in hernial sac and white arrow showing undescended right testis.

40 year male presented to emergency with chief complaints of irreducible swelling in the right inguinoscrotal region, vomiting and not able to pass stool and flatus for 4 days. The swelling in the right inguinal region was present for 2 months which was painless, reducible and non-progressive in size but since 4 days, it was irreducible and was associated with pain over swelling and vomiting. The pain was acute in onset, dull in character, non-radiating with no aggravating or relieving factors. Vomiting was non projectile, non-blood stained, feculent, foul smelling, 4–5 episodes per day and copious in amount. He was not able to pass stool and flatus. So during these 4 days, he used to strain multiple times a day for defecation. There was no history of chronic cough, trauma to the abdomen or groin, any medical comorbidities or surgical intervention in the past. He used to smoke and consume alcohol for 20 years. On examination, there was swelling of 5 × 3 cm^2^ at right inguinoscrotal region which was firm, tender and irreducible. The right testes was not palpable separately whereas the left testes was palpable. Abdominal examination showed features of peritonitis. Nasogastric tube and Foley's catheterization along with fluid resuscitation were done. On nasogastric tube insertion, 300 ml feculent collection was noted. Ultrasonography showed a fascial defect of 2.5 cm in the right inguinal region through which bowel and omentum were protruding with normal vascularity and peristalsis of bowel. It also showed right undescended testis, lying in the right inguinal canal alongside the hernia content with dilated bowel loops. X-ray abdomen erect and supine showed dilated bowel loops with air-fluid level. On inguinoscrotal exploration, the hernia sac contained an ileal loop which was viable but spontaneously transected (Fig. 1). The bowel loop was not dusky, and had normal peristaltic movement and vascularity. The nearby mesentery and mesenteric vessels looked grossly normal which excluded ischemia induced transection. 10 ml of toxic fluid was present within the sac. So, hand-sewn ileoileal anastomosis with darning repair with right orchidectomy was done. The patient was discharged on 6th post-operative day without any complications.

## 3 Case discussion

Intraluminal pressure of 150–260 mm of Hg is required for the transection of bowel. Blunt trauma to the inguinal region in an inguinal hernia patient compresses the afferent and efferent bowel loops creating an intraluminal pressure of 300 mm of Hg which exceeds the intraluminal pressure of 150–260 mm of Hg and hence leads to bowel transection [7].

Bowel transection following non-traumatic events like coughing or straining is rare [8]. Straining induced bowel transection, as seen in our case, has not been reported. In incarcerated hernia, bowel loops are edematous, and repeated episodes of straining during defecation can lead to an elevation of intraluminal pressure up to 230 mm of Hg, which is enough to cause transection of edematous bowel [9]. Similar report of bowel transection in incarcerated hernia has been reported by Hakiman et al. but it was cough induced rather than straining induced. Cough reflex can lead to development of maximum intraluminal pressure of 110 mm of Hg. Although this pressure is not enough to cause transection of healthy non-edematous bowel, it can cause transection in edematous bowel due to long standing incarcerated hernia [8].

In a study conducted by Loftus et al., mesh repair was done in 50 patients with incarcerated hernia with CT evidence of toxic fluid in the hernia sac. Out of them, more than 50 % of patients with evidence of toxic fluid in hernia sac, developed surgical site infection [10].

According to 2017 update of WSES guidelines for emergency repair of complicated abdominal wall hernia, mesh repair is recommended for those cases of incarcerated or strangulated hernia, where the colour of bowel reverts back to normal on hot mopping. Mesh repair can be performed even in those cases of strangulated or incarcerated hernia, where bowel resection is done but there is no gross spillage of bowel contents in the surgical field. However, in the setting of bowel resection with gross spillage of bowel contents in the surgical field, synthetic polypropylene mesh repair is not recommended. In such cases, suture based repair (like darning repair/nylon darn technique) or the use of biological or polyglactin mesh is recommended [11].

In a systematic review and meta-analysis done by Marcolin et al. in 2023, 12,402 patients of incarcerated or strangulated groin hernia, from 20 observational studies and 4 randomized control trials, were analyzed. It was observed that mesh repair in incarcerated or strangulated hernia led to reduced recurrence, operative time and length of hospital stay without causing any significant increase in the rate of surgical site infection, mortality or post- operative complications (seroma formation, ileus, urinary retention). However, in the group undergoing bowel resection due to an incarcerated or strangulated hernia, mesh repair led to a higher risk of surgical site and mesh infection. This study concluded that it's better to do mesh repair, in those cases of incarcerated or strangulated hernia, where the colour of bowel reverts back to normal on hot mopping and bowel resection is not required. However, in the cases where bower resection is required, it's better to perform suture based repair like darning repair (as done in our case) but avoid polypropylene synthetic mesh repair [12].

The Nylon Darn technique was first introduced by Abrahamson and later on, it was popularized by Moloney in 1948. In this technique, conjoint tendon is approximated with shelving edge of inguinal ligament using monofilament nylon without forcefully bringing the tissue together [13]. The repair is initiated from pubic tubercle, continued beyond deep inguinal ring and is reverted back to pubic tubercle [14]. Although nylon was used in the original technique of darning developed by Moloney, use of polypropylene suture has been found in literatures [15].

The incidence of undescended testis in preterm is 30 % whereas in full term it ranges from 2 % to 5 % [16]. It drops to 1 % after the age of 3 months of birth. Untreated undescended testis is at higher risk of developing malignancy after 10 years of age [17]. Similarly, restoration of fertility is not seen even if orchidopexy is done in adults. Decrease in number of spermatogonial cells, testicular atrophy and interstitial hyperplasia progress in cryptorchidism after 1.5 years of age [18]. Due to these reasons, orchidectomy is advised if unilateral cryptorchidism is detected in adult life [16] (as seen in our case).

## 4 Conclusion

In incarcerated hernia, since the bowels are edematous, even non traumatic events like repeated episodes of coughing or straining can cause transection of bowel. In the setting of bowel resection, it's better to perform suture based repair like darning repair and avoid mesh repair as there is probability of spillage of bowel content making the surgical field contaminated. Undescended testis if found in an adult, orchidectomy is always advised as compared to orchidopexy.

## Author contribution

1. Constructing hypothesis for the manuscript: Rahul Jha, Bijay Raj Bhatta, Samrat Shrestha
2. Planning methodology to reach the conclusion: Rahul Jha, Ramesh Prasad Upadhayay, Bijay Raj Bhatta, Samrat Shrestha, Rishika Prasad
3. Organizing and supervising the course of the article and taking responsibility: Rahul Jha, Rishika Prasad, Ramesh Prasad Upadhayay
4. Patient follow-up and reporting: Rahul Jha, Ramesh Prasad Upadhayay, Rishika Prasad, Bijay Raj Bhatta
5. Logical interpretation and presentation of the results: Samrat Shrestha, Rishika Prasad, Bijay Raj Bhatta
6. Construction of the whole or body of the manuscript: Rahul Jha, Ramesh Prasad Upadhayay, Rishika Prasad, Bijay Raj Bhatta, Samrat Shrestha
7. Reviewing the article before submission not only for spelling and grammar but also for its intellectual content: Rahul Jha, Bijay Raj Bhatta, Samrat Shrestha, Rishika Prasad, Ramesh Prasad Upadhayay

## Informed consent

Written informed consent was obtained from the patient for publication of this case report and accompanying images. A copy of the written consent is available for review by the Editor-in-Chief of this journal on request.

## Ethical approval

Ethical approval is waived at our institution (National Academy of Medical Science, Bir Hospital) and this study was exempt from ethical approval at our institution, as this paper reports a single case that emerged during a normal surgical case report.

## Guarantor

Rahul Jha accepts full responsibility for the work and/or the conduct of the study, has access to the data, and controls the decision to publish.

## Research registration number

Not applicable.

## SCARE guideline

The work has been reported in line with the SCARE criteria [6].

## Funding

This research did not receive any specific grant from funding agencies in the public, commercial, or not-for-profit sectors.

## Conflict of interest statement

No conflict of interest.
